# EchinoDB, an application for comparative transcriptomics of deeply-sampled clades of echinoderms

**DOI:** 10.1186/s12859-016-0883-2

**Published:** 2016-01-22

**Authors:** Daniel A. Janies, Zach Witter, Gregorio V. Linchangco, David W. Foltz, Allison K. Miller, Alexander M. Kerr, Jeremy Jay, Robert W. Reid, Gregory A. Wray

**Affiliations:** 1 Department of Bioinformatics and Genomics, University of North Carolina at Charlotte, 9201 University City Blvd, Charlotte, NC 28223-0001 USA; 2 Department of Biological Sciences, Louisiana State University, Baton Rouge, LA 70803 USA; 3 Marine Laboratory, University of Guam, University Dr, Mangilao, 96923 Guam; 4 Department of Biology, Duke University, Durham, NC 27708 USA

**Keywords:** Echinoderm, RNA-Seq, Database, Ortholog, Paralog, Transcriptome, Phylogeny, Developmental biology, Neuroscience, Gene family

## Abstract

**Background:**

One of our goals for the echinoderm tree of life project (http://echinotol.org) is to identify orthologs suitable for phylogenetic analysis from next-generation transcriptome data. The current dataset is the largest assembled for echinoderm phylogeny and transcriptomics. We used RNA-Seq to profile adult tissues from 42 echinoderm specimens from 24 orders and 37 families. In order to achieve sampling members of clades that span key evolutionary divergence, many of our exemplars were collected from deep and polar seas.

**Description:**

A small fraction of the transcriptome data we produced is being used for phylogenetic reconstruction. Thus to make a larger dataset available to researchers with a wide variety of interests, we made a web-based application, EchinoDB (http://echinodb.uncc.edu). EchinoDB is a repository of orthologous transcripts from echinoderms that is searchable via keywords and sequence similarity.

**Conclusions:**

From transcripts we identified 749,397 clusters of orthologous loci. We have developed the information technology to manage and search the loci their annotations with respect to the Sea Urchin (*Strongylocentrotus purpuratus*) genome. Several users have already taken advantage of these data for spin-off projects in developmental biology, gene family studies, and neuroscience. We hope others will search EchinoDB to discover datasets relevant to a variety of additional questions in comparative biology.

**Electronic supplementary material:**

The online version of this article (doi:10.1186/s12859-016-0883-2) contains supplementary material, which is available to authorized users.

## Background

In many studies focused on using transcriptomics to reconstruct phylogenetic trees, most of the RNA-Seq data are filtered out and do not end up in a matrix for phylogenetic tree search. However the data not used in phylogenetics can be valuable for other purposes such as developmental biology [1], gene family studies [2, 3], neuroscience [4] as well as new ideas that will come from the community. Thus we make much of our transcriptome data freely available via an application called EchinoDB (http://echinodb.uncc.edu). The data can be accessed via text or sequence similarity searches.

Echinoderms are an exclusively marine phylum of deuterostome animals that share a deep common ancestor with chordates. The body plans of extant Echinoderms range from stalked, flower-like sea lilies, to ambulatory and stellate starfish and brittle stars, to soft-bodied sea cucumbers, to spiked, armored and globose sea urchins, to flat sand dollars. The benthic adult forms of these diverse animals share a water-vascular system in which a central coelomic ring extends to form five (and sometimes more) radial canals bearing tube feet. In contrast with the pentaradial form of benthic adults, most echinoderm larvae are bilaterally symmetric and a drastic metamorphosis is required to form the adult body. The diversity of echinoderm life cycles, anatomy and their shared ancestry with chordates make echinoderms important models in a variety of comparative disciplines. In this project we provide a means for investigators to find gene families of interest to their questions across biology.

## Construction and content

RNA from muscle tissues samples (adult tube feet, pinnules or body wall) from 42 Echinoderm specimens (Additional file 1) was extracted using a Qiagen miRNEasy kit. An Agilent Bioanalyzer 2100 ver. 2.6 was used for quality control prior to library preparation. Samples were then submitted to the Duke Institute for Genome Science and Policy for library preparation with an Illumina TruSeq RNA kit, followed by RNA-Seq sequencing on an Illumina Hiseq 2000 platform (100 BP, paired end). Reads for each of the samples were filtered by quality score (cutoff threshold > Q20) by fastxtrimmer, Illumina adapters were then removed by fastxclipper, both components of the fastx toolkit [5].

RNA-Seq produced a total of 2.3 billon raw reads. Following trimming and adapter removal, 2.1 billion reads remained, an overall reduction of approximately 11 %. The sample from *Pisaster ochraceus* had the most reads at 88,987,394. The *Cheiraster* sp. sample had the least amount of reads at 30,190,658. The sample from *Promachocrinus kerguelensis* had the most reads removed with a decrease of nearly 19 %. On the other end of the spectrum, the sample from *Gephyrocrinus messingi* had the least amount of reads removed at a reduction of 3.64 %. There was no observed correlation between taxonomic level and read count. *De novo* assembly of contigs was then performed using Trinity [6] on a high memory compute cluster using 500 GB of RAM and 24 CPUs.

Contigs for each sample were conceptually translated into peptides using Transdecoder [7] and the PFAM-B protein family database [8] (minimum protein length = 100). Each translated contig was compared to all other contigs in order to discover orthologous clusters using OrthoMCL which uses BLASTP [9]. To provide an initial annotation to the assembled contigs for each OrthoMCL cluster, 24,829 protein sequences for *Strongylocentrotus purpuratus* were downloaded from NCBI [10] and included in the OrthoMCL clustering. Most of these species have never been sequenced by any high throughput technology except for *Strongylocentrotus purpuratus.* This provided an opportunity to compare our *Strongylocentrotus purpuratus* contigs derived from the transcriptome to the publically available genome data for *Strongylocentrotus purpuratus.* We compared the *Strongylocentrotus* RefSeq dataset to our nucleotide contigs with BLASTN and found that 91.6 % of our contigs formed high scoring pairs (E-value 1e-10) with members of the RefSeq dataset.

EchinoDB is written using the Go programming language and Revel web framework, and is serviced by the NGINX web server. NGINX allows for load balancing and transparent server redirections in the web application. The redirection allows a single domain name to serve both the EchinoDB keyword search functionality and a BLAST (sequence similarity) interface using SequenceServer [11]. All of the relational data and clusters are stored in a PostgreSQL database, and all sequence files are stored and indexed by BLAST on the local file system.

## Utility and discussion

**Fig. 1:**
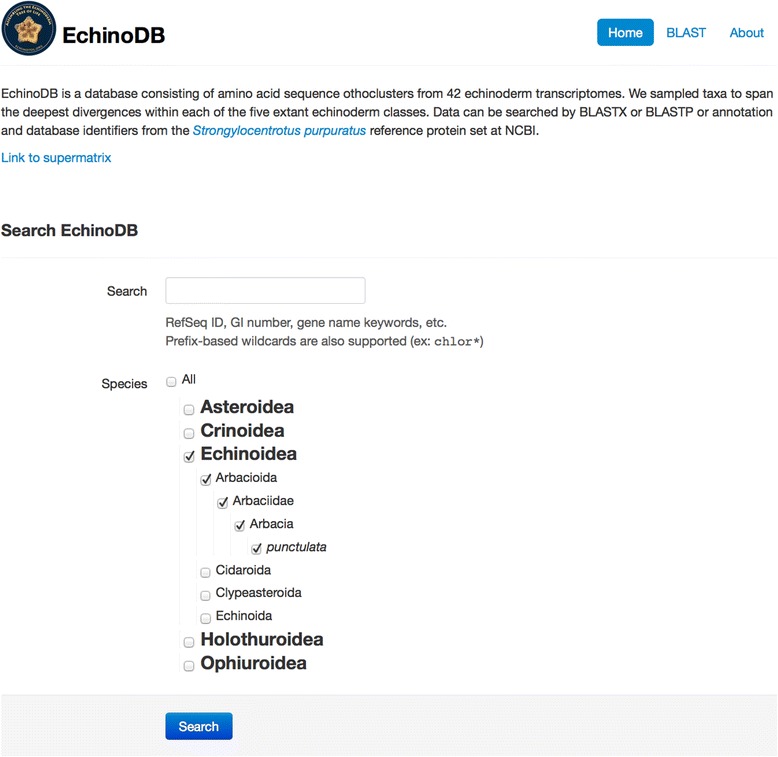
Screenshot of the http://echinodb.uncc.edu landing page. This user has prepared to search a keyword against data for *Arbacia punctulata*. Alternatively the user could search against all species or a taxonomically defined subset

**Fig. 2:**
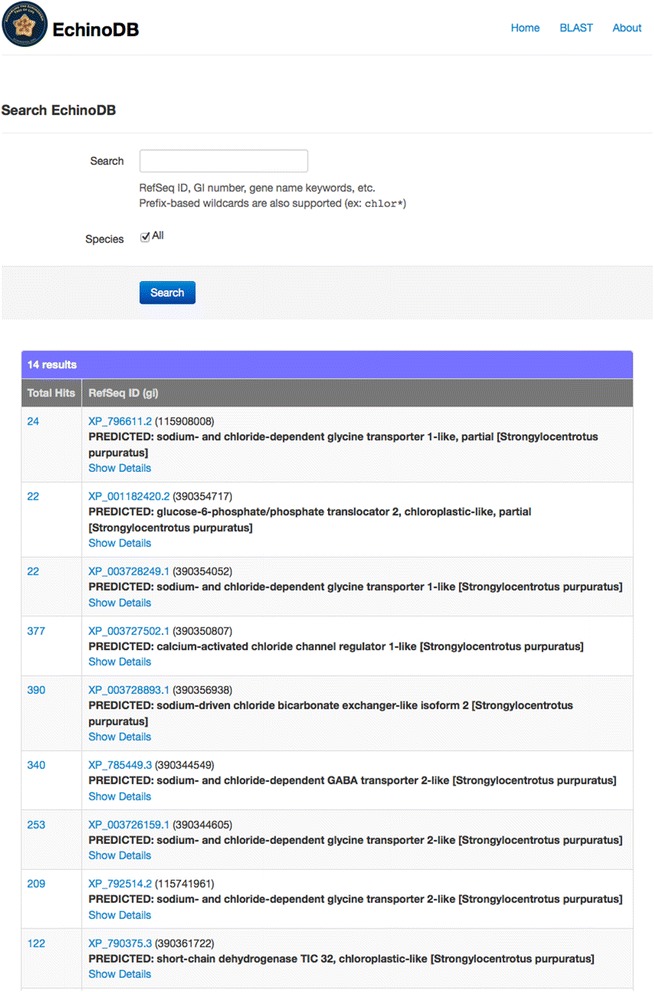
Screenshot of part of the http://echinodb.uncc.edu page showing a result for the wildcard keyword search “chlor*” against all the data

The EchinoDB user is greeted with a simple text box for searching fields such as RefSeq ID, GI number, gene name, or other keywords. Prefix-based wildcards are also supported (e.g.: chlor*). Hierarchical taxonomy selection allows the user to direct the text search against all the specimens or a subset of specimens scoped by zoological classification (Fig. 1). Results are returned in a table with two columns (Fig. 2). Each row of the table represents an orthocluster. The orthoclusters contain putative orthologous and paralogous sequences. The right cell of each row displays the *Strongylocentrotus purpuratus* protein RefSeq id and narrative description of the gene. The RefSeq id is linked to NCBI’s Entrez. The left cell of each row contains the number of sequences in the orthocluster that hit (i.e. exhibit similarity as defined by blast E-value < 1e-25) to a *Strongylocentrotus purpuratus* protein RefSeq (Fig. 2)*.* That integer for the number of hits is linked such that when clicked the user can see all the members of the orthocluster and basic statistics on each contig including: The consensus length, the number of reads that formed that contig, and the average number of reads per kilobase. In this view, the user can see the amino acid sequence data and view the conceptual translation. The user can download from EchinoDB their choice of nucleotide gene sequences, coding sequences, peptide sequences, or all the cluster sequences in a file compressed with zip. Alternatively, the user can follow links from EchinoDB to corresponding Refseq and bioproject resources in NCBI.

**Fig. 3:**
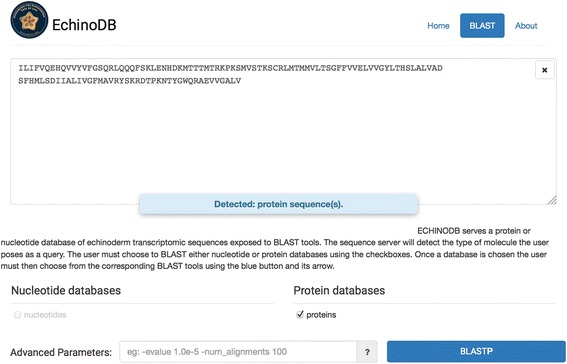
Screenshot of the http://echinodb.uncc.edu page showing that the BLAST interface can detect a protein query

In addition to keyword searching and to provide a means to search for data across the entire set of transcriptomes, we also provide a BLAST interface as implemented by SequenceServer (Fig. 3). In this field, the user can provide an arbitrary amino acid or nucleotide sequence and SequenceServer will suggest the appropriate BLAST program and parameters for the search. SequenceServer detects the sequence type of the user inputs and suggests default parameters. The user selects either the nucleotide or protein database, adjusts the default parameters if desired, and clicks the blastp or blastn button. After calculation, BLAST results are returned in a standard, easily recognizable format to anyone familiar with the tool. The user can select one or more high scoring pairs (HSP) for download (current limit is 500) in XML format for later processing and downstream analyses.

There is one well-annotated echinoderm genome, *Strongylocentrotus purpuratus*, in the public domain. As a result, the keyword search interface to our database is currently searchable by *Strongylocentrotus purpuratus* RefSeq ID, GI number, gene name, or other text in the annotation of this species. *Strongylocentrotus purpuratus* RefSeq proteins can participate in the formation of a cluster but are not required to form a cluster.

## Conclusions

This is the first large collection of data for transcriptomes sampled across the Phylum Echinodermata, including rare and deep-sea taxa. Given the ancient evolutionary history of the phylum, it is crucial to have a resource that can provide insight via well-designed taxonomic comparisons. In contrast, other efforts have focused on *Strongylocentrotus* and a handful of easy-to-collect echinoderms and outgroups [12, 13].

Several users have already taken advantage of the data in EchinoDB for spin-off projects across taxa and disciplines. Developmental biologists have used EchinoDB data to study variation in skeletogenic proteins among ophiuroid and echinoid echinoderms [1]. Biologists interested in gene families have used EchinoDB data to discover echinoderm hemoglobins related to the vertebrate neuroglobin and cytoglobins [2]. Another group has used EchinoDB data to uncover variants within the tissue inhibitors of metalloproteinases gene family. These genes are involved in the physiology of mutable collagenous tissue in echinoderms, especially holothuroids which are known to have a wide range body elasticity and can eviscerate [3]. Neuroscientists have used EchinoDB data to study variation in echinoderm neuropeptide precursors, known as SALMFamides. This work has opened a new line of research for the role of the SALMFamides variants extra-oral feeding in asteroids [4]. We hope that users will find our application easy to use and the echinoderm tree of life transcriptome data useful in a variety of endeavors.

## Availability and requirements

Use of http://echinodb.uncc.edu, its data, and source code http://zwitter1@bitbucket.org/bioservices/echinodb.git#_blank are unrestricted for use by academic and commercial researchers.

## Additional file

Additional file 1:
**Table of 42 echinoderm specimens used for RNA-seq data that are contained in** **http://echinodb.uncc.edu**. The BJ number is an internal reference code. The voucher number represents where any residual tissues and metadata are stored. RAW indicates the number of raw reads produced by Illumina sequencing. Quality filter and adapter removal indicates the number of reads remaining following fastxtoolkit quality filter of Q score > 20 and removal of adapter regions. Percent reads remaining indicates the fraction of raw reads retained after quality filtering and adapter removal. Percentage Reads removed indicates the fraction of reads removed by quality filtering and adapter removal from the raw reads. Number of Amino Acid Sequences Participating in Orthologous Clusters indicates number of contigs for each species that participated in orthoclusters. Note that contigs may be partially overlapping and redundant. NCBI BioProject Accession number indicates where the contigs have been submitted to NCBI (note the orthoclusters only exist on http://echinodb.uncc.edu). (XLSX 33 kb)
